# Subclinical Cardiovascular Damage and Fat Utilization in Overweight/Obese Individuals Receiving the Same Dietary and Pharmacological Interventions

**DOI:** 10.3390/nu6125560

**Published:** 2014-12-01

**Authors:** Tiziana Montalcini, Theodora Lamprinoudi, Gaetano Gorgone, Yvelise Ferro, Stefano Romeo, Arturo Pujia

**Affiliations:** 1 Clinical Nutrition Unit, Department of Medical and Surgical Science, University Magna Grecia, Catanzaro 88100, Italy; E-Mails: tlamprinoudi@hotmail.it (T.L.); ggorgone58@libero.it (G.G.); ferro@unicz.it (Y.F.); romeo@unicz.it (S.R.); pujia@unicz.it (A.P.); 2 Sahlgrenska Center for Cardiovascolar and Metabolic Research, Department of Molecular and Clinical Medicine, University of Gothenburg, Gothenburg 40530, Sweden

**Keywords:** cardiac remodeling, obesity, nutrient utilization, echocardiography, carotid, fat utilization

## Abstract

Subclinical organ damage precedes the occurrence of cardiovascular events in individuals with obesity and hypertension. The aim of this study was to assess the relationship between fuel utilization and subclinical cardiovascular damage in overweight/obese individuals free of established cardiovascular disease receiving the same diet and pharmacological intervention. In this retrospective study a total of 35 subjects following a balanced diet were enrolled. They underwent a complete nutritional and cardiovascular assessment. Echocardiography and ultrasonography of the carotid arteries was performed. The respiratory quotient (fuel utilization index) was assessed by indirect calorimetry. A total of 18 had left ventricular concentric remodeling, 17 were normal. Between these two groups, a significant difference of intima-media thickness was showed (*p* = 0.015). Also a difference of respiratory quotient was shown with the highest value in those with remodeling (*p* = 0.038). At univariate and multivariate analysis, cardiac remodeling was associated with respiratory quotient (RQ) (*p* = 0.04; beta = 0.38; SE = 0.021; *B* = 0.044). The area under the receiver operating characteristic (ROC) curve for respiratory quotient to predict remodeling was 0.72 (SE = 0.093; *p* = 0.031; RQ = 0.87; 72% sensitivity, 84% specificity). The respiratory quotient is significantly different between those participants with and without cardiac remodeling. Its measurement may help for interpreting the (patho)physiological mechanisms in the nutrients utilization of obese people with different response to dietary or pharmacological interventions.

## 1. Introduction

It is well known that myocardial tissue can increase the utilization of glucose in response to the increased workload [1].It has been shown that mechanism for shifting from fat to glucose utilization is advantageous, since carbohydrates are more efficient substrates than lipids and they can protect the heart from injuries, like cardiac ischemia and reperfusion injury [2]. Alterations of substrate metabolism have been reported in several models of left ventricular hypertrophy (LVH) and heart failure leading to the hypothesis that a shift away from fatty acid (FA) use toward glucose contributes to the preservation of efficiency in cardiac remodeling [2,3,4]. The myocardial metabolic response associated with many heart diseases has been extensively investigated [1,2,3,4] but not in subjects with early signs of organ damage. Furthermore, in most of these studies the respiratory quotient (RQ) assessment, an index of nutrient utilization measured with indirect calorimetry [5], has not been used. High RQ is associated with a high rate of subsequent weight gain [5], hypertension [6] and increased Carotid Intima-Media Thickness (CIMT) [7], a well-known predictor of cardiovascular events [8] suggesting that subjects with these conditions tend to burn more glucose but less fat. Indeed major values of CIMT are present in concentric LVH [9], therefore it is possible to assume a relationship between RQ and cardiac remodeling. Since the development of subclinical organ damage precedes and predicts the occurrence of cardiovascular events in obese, as well as hypertensive patients, the aim of this study was to assess whether there was a relationship between RQ and both detrimental cardiac chamber remodeling and carotid intima-media thickness in overweight/obese individuals free of established cardiovascular disease (CVD). This information may be relevant for interpreting the (patho)physiological mechanism of nutrients utilization that may occur in subjects affected by obesity receiving dietary or pharmacological interventions and for identifying new therapeutic strategies.

## 2. Experimental Section

This is a population-based retrospective study, performed from July 2013 until October 2013, in white overweight/obese subjects attending the outpatient Dietetic Clinic of the University Magna Grecia in Catanzaro. We used baseline data from the Clinic’s database to recall and recruit participants over 35 years old among those who had received a dietary intervention during the last year. We excluded individuals with clinical evidence of debilitating diseases, such as chronic illness (cancer, severe renal failure, sever liver insufficiency and chronic obstructive pulmonary disease), thyroid dysfunction and established cardiovascular disease (myocardial infarction, stroke, peripheral artery disease), as well as subjects taking anti-obesity medications, psychotropic drugs and chronotropic agents.

We therefore recalled about 50 overweight/obese subjects who underwent a medical interview and examination so that we could collect all the known classical CVD risk factors and perform nutritional and cardiovascular assessments.

The following criteria were used to define the distinct cardio-metabolic risk factors; diabetes: fasting blood glucose ≥ 126 mg/dL or antidiabetic treatment; hyperlipidemia: total cholesterol > 200 mg/Dl and/or triglycerides > 200 mg/dL or lipid lowering drugs use; hypertension: systolic blood pressure ≥ 140 mmHg and/or diastolic blood pressure ≥ 90 mmHg or antihypertensive treatment; overweight: 25 kg/m^2^ ≤ BMI < 30 kg/m^2^; obesity: body mass index (BMI) ≥ 30 kg/m^2^; smoking: current smokers [10,11]. Written informed consent was obtained. The protocol was approved by local ethical committee at the Azienda Mater Domini University Hospital (projects codes 2013-1/CE). The investigation conforms to the principles outlined in the Declaration of Helsinki.

### 2.1. Blood Pressure Measurement

The measurement of the systemic blood pressure (BP) of both arms was obtained by a mercury sphygmomanometer (systolic blood pressure (SBP) and diastolic blood pressure (DBP)) as previously described [12]. Clinic BP was obtained in supine patients, after 5 min of quiet rest. A minimum of three BP readings were taken using an appropriate BP cuff size (the inflatable part of the BP cuff covered about 80 percent of the circumference of upper arm).

#### 2.1.1. Biochemical Evaluation

Venous blood was collected after fasting overnight into vacutainer tubes (Becton & Dickinson, Plymouth, England) and centrifuged within 4 h. Serum glucose, creatinine, total cholesterol, high density lipoprotein (HDL)-cholesterol, triglycerides, uric acid were measured with Enzymatic colorimetric test. Serum calciumwas evaluated with a colorimetric assay according to Schwarzenbach, using *O*-cresolphthalein-complexone. Quality control was assessed daily for all determinations.

#### 2.1.2. Anthropometric and Nutritional Intake Measurements

All tests were performed after a 12 h overnight fasting. Before tests, participants had no caffeinated beverages between their evening meal and the conclusion of the tests on the examination’s morning. Body weight was measured before breakfast with the subjects lightly dressed, subtracting the weight of clothes. Body weight was measured with a calibrated scale and height measured with a wall-mounted stadiometer. BMI was calculated with the following equation: weight (kg)/height (m)^2^. Waist circumferences and hip circumferences (WC and HC) were measured with a nonstretchable tape over the unclothed abdomen at the narrowest point between the costal margin and iliac crest and over light clothing at the level of the widest diameter around the buttocks, respectively, as described in the past [13].

The participant’s nutritional intake was calculated using the nutritional software MetaDieta 3.0.1 (Metedasrl, San Benedetto del Tronto, Italy). All subjects following a solid-food diet that supplied 50%–55% of the calories as carbohydrate, 18%–20% as protein and no more than 30% as fat during at least the previous three months and who were also weight stable were enrolled.

Bioelectrical impedance analysis (BIA) (BIA-101, Akernsrl, Florence, Italy) was performed to estimate the Total Body Water (TBW), Fat Mass (FM), Muscle Mass (MM), total Fat-Free Mass (FFM), and extra cellular water (ECW) [14].

Fasting RQ and the Resting Metabolic Rate (RMR) were measured with the participants in their postabsorptive state in a sedentary position. Respiratory gas exchange was measured by Indirect Calorimetry using the open circuit technique between the hours of 7 a.m. and 8:30 a.m. after 48 h abstention from exercise. The Indirect Calorimetry instrument (Viasys Healthcare, Hoechberg, Germany) was used for all measurements. The participant rested quietly for 30 min in an isolated room with temperature controlled (21–24 °C) environment. The subject was then placed in a ventilated hood for at least 30 min, until steady state was achieved. Criteria for a valid measurement was a minimum of 15 min of steady state, with steady state determined as less than 10% fluctuation in minute ventilation and oxygen consumption and less than 5% fluctuation in RQ. RQ was calculated as CO_2_ production/O_2_ consumption [7,15]. In a subgroup of 6 participants, we repeated the RQ measurements on different days. The interassay coefficient of variation (CV) is calculated from the formula:

CV% = (Standard Deviation (SD) of Mean × 100)/Mean (1)

The interassay CV resulted 6.77% (SD of the means of the duplicates—0.06, Grand Mean of the duplicates—0.886).

### 2.2. Cardiovascular Assessment

#### 2.2.1. Echocardiographic Evaluation

The M-Mode, 2D, and Doppler echocardiographic evaluations were performed with the subjects in lateral position with a 2- to 3.2-MHz transducer in harmonic imaging interfaced with a commercially available echocardiographic machine (Advanced Technology Laboratories—ATL, High Definition Imaging—HDI 5000, Bothell, WA, USA). Left ventricular end-diastolic and end-systolic diameters and interventricular septum and posterior wall diastolic thickness were identified in the parasternal long-axis view during M-mode tracing [16]. Left ventricular mass (LVM) in grams was calculated by the Devereux formula (using ventricular end-diastole measurements) [17]. Also, the left atrial dimension and function were measured as previously described [18]. All the examinations were performed by the same ultrasonographer blinded to the clinical information (operator: T. Lamprinoudi).

#### 2.2.2. Left Ventricular Geometric Pattern and Systolic Function

LVM index (LVMi) was calculated as LVM divided by body surface area (BSA) [19] as follows:

LVMi = LVM/(0.0001 × 71.84 × (weight-kg) 0.425 × (height-m) 0.725) (2)

A normal left ventricular mass was defined as a mass <125 g/m^2^ in both men and women [19]. However, the normal values for LV mass in men and women indexed for the body surface area were considered [20]. Subjects were classified into two groups: normal left ventricular geometry and left ventricular concentric remodeling (LVCR), the second defined by the thickness of the septum or posterior wall divided by left ventricular radius equal or more than 0.45 [21,22]. Left ventricular end-diastolic and end-systolic volumes and the ejection fraction were also calculated [23].

### 2.3. Carotid Arteries Assessment

The subjects underwent B-mode ultrasonography of the extracranial carotid arteries by use of a duplex system (a high-resolution ultrasound instrument ATL, HDI 5000 with a 5- to 12-MHz linear array multifrequency transducer). All the examinations were performed by the same ultrasonographer blinded to clinical information (Gaetano Gorgone). All patients rested in the supine position for at least 10 min before the study and were kept in this position during the procedure. ECG leads were attached to the ultrasound recorder for on-line continuous heart rate monitoring. The right and left common (CCA) and internal carotid arteries (including bifurcations) were evaluated with the head of the subjects turned away from the sonographer and the neck extended with mild rotation. The IMT, defined as the distance between the intimal-luminal interface and the medial—adventitial interface, was measured as previously described [7,24]. Briefly, in posterior approach and with the sound beam set perpendicular to the arterial surface, 1 cm from the bifurcation, three longitudinal measurements of IMT were completed on the right and left common carotid arteries far-wall, at sites free of any discrete plaques. The mean of the three right and left longitudinal measurements was then calculated. Then, we calculated and used for statistical analysis the mean CIMT between right and left CCA. The coefficient variation of the methods was 3.3%. To evaluate artery diameters, images were magnified, whereas depth and gain settings were set to optimize the image of the vessel wall, in particular, the media-adventitia interface (“m” line). The end-diastolic diameter of the vessel, defined as the distance between near-wall and far-wall junctions of the media and adventitia, was measured over four cardiac cycles with the use of digital calipers and the average was then calculated.

### 2.4. Statistical Analysis

Data are reported as mean ± (SD). A chi square test was performed to analyze the prevalence of the risk factors and a *t*-test was performed to compare the means between subjects with and without cardiac chamber remodeling.

The Pearson correlation was used to identify the variables correlated to the cardiac chamber remodeling given that the continuous variables were normally distributed. The Multivariate linear regression analysis was used to test the association between the cardiac chamber remodeling and the confounding variables selected among all that in the univariate analysis correlated with the cardiac remodeling having a *p* < 0.1. Furthermore, the area under the receiver operating characteristic (ROC) curve was used to analyze the capacity of RQ to predict the presence of cardiac chamber remodeling. Significant differences were assumed to be present at *p* < 0.05 (two-tailed). All comparisons were performed using SPSS 20.0 for Windows (IBM Corporation, New York, NY, United States). 

## 3. Results

We enrolled 35 overweight/obese subjects (female *n* = 27; 12 were in menopause). A total of 18 had LVCR and 17 had normal geometry (relative wall thickness 0.54 ± 0.09 cm and 0.35 ± 0.04 cm respectively), none had LV hypertrophy (LVH).

Table 1 shows the prevalence of the CV risk factors according to the presence of LVCR. A total of 18 subjects used antihypertensive agents. Between subjects with and without LVCR, the use of these medications (including different types) was not significantly different (Table 1). None of them used lipid-lowering or antidiabetics medications.

**Table 1:** Cardiovascular risk factors prevalence according to the presence of left ventricular concentric remodeling (LVCR).

Variables	Normal Geometry	LVCR	*p*
Male sex (%)	23.5 (4)	26 (5)	0.58
Diabetes (%)	5.9	5.9	0.75
Hypertension (%)	41.2	43.8	0.58
Dyslipidemia (%)	31.3	33.3	0.60
Smoking (%)	11.7	6.6	0.58
Menopausal status (%)	50	50	0.34
Angiotensin-converting-enzyme inhibitor (%)	53.8	46.2	0.61
Diuretics (%)	40	60	0.52

**Figure 1:**
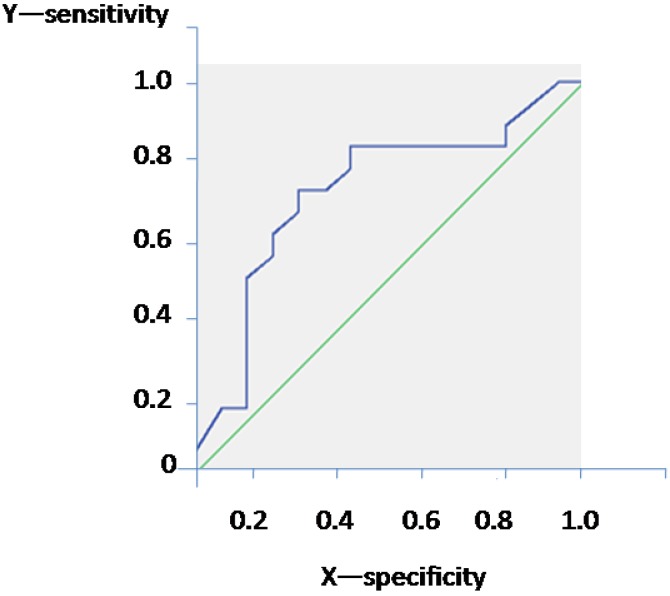
The area under the receiver operating characteristic (ROC) curve for Respiratry Quotienty (RQ) to predict the presence of left ventricular concentric remodeling.

Table 2 shows the general and cardiovascular characteristics of the population according to the presence of LVCR. A significant difference in the CIMT was found between groups (*p* = 0.015). Furthermore, CIMT resulted in significant difference between participants with and without a high RQ value (more than 0.85) [5,6] (0.75 ± 0.20 and 0.58 ± 0.1 respectively; *p* = 0.042). A significant difference in the RQ was found with the highest value in the subjects with LVCR (*p* = 0.038, Table 2). In the univariate analysis, LVCR was associated with the following factors: glucose (*r* = 0.40; *p* = 0.01), triglycerides (*r* = 0.29; *p* = 0.08), hypercholesterolemia (*r* = 0.31; *p* = 0.09) and RQ (*r* = 0.40, *p* = 0.02); the multivariate analysis confirmed an association only between LVCR and RQ (beta = 0.42; SE = 0.35; *B* = 0.87, *p* = 0.02; table not shown). The area under the ROC curve for RQ to predict the presence of LVCR was 0.72 (SE = 0.093; *p* = 0.031; Table 3). The RQ equal to 0.875 achieved satisfactory sensitivity (72%) and specificity (84%) (Figure 1).

**Table 2:** General and cardiovascular characteristics of the population according to the presence of left ventricular concentric remodeling (LVCR).

Variables	Normal Geometry	LVCR	*p*
Age (years)	50.06 ± 11	54.06 ± 10	0.297
RMR (joule)	5796 ± 715	6137 ± 1137	0.195
RQ	0.85 ± 0.05	0.89 ± 0.05	0.038
BMI (kg/m^2^)	32.56 ± 5	32.78 ± 7	0.924
WC (cm)	99.38 ± 10	102.87 ± 15	0.477
HC (cm)	105.97 ± 10	109.37 ± 11	0.274
TBW (Lt)	38.53 ± 7	40.06 ± 8	0.615
ECW (Lt)	17.04 ± 3	17.85 ± 3	0.514
FFM (kg)	52.09 ± 10	53.46 ± 12	0.755
MM (kg)	35.01 ± 7	36.22 ± 10	0.734
FM (kg)	28.47 ± 10	33.07 ± 12	0.297
Glucose (mmol/L)	5.12 ± 0.4	5.60 ± 1.3	0.177
Creatinine (μmol/L)	64.2 ± 8	66 ± 17	0.931
T Cholesterol (mmol/L)	5.89 ± 1.68	5.79 ± 1	0.835
HDLCholest (mmol/L)	1.43 ± 0.28	1.42 ± 0.41	0.956
LDLCholest (mmol/L)	3.91 ± 1.47	3.63 ± 0.98	0.521
Triglycerides (mmol/L)	1.21 ± 0.5	1.73 ± 1	0.083
Calcium (mg/dL )	9.51 ± 0.39	9.42 ± 0.34	0.508
Uric Acid (μmol/L)	255.7 ± 59	296.2 ± 59	0.309
SBP (mmHg)	133.18 ± 14	128.41 ± 31	0.579
DBP (mmHg)	86.41 ± 8	80.82 ± 6	0.036
HR (b/m)	71.71 ± 7	73.06 ± 7	0.607
Mean CIMT (mm)	0.57 ± 0.11	0.80 ± 0.19	0.015
LVMI (g/m^2^)	72.51 ± 11	77.14 ± 11	0.243
AR (mm)	31.06 ± 3	32.06 ± 2	0.315
LAD (mm)	33.94 ± 3	34 ± 4	0.965
LVend-dias diam(mm)	47.35 ± 3	42.39 ± 5	0.004
LV end-syst diam(mm)	32.12 ± 3	27.94 ± 5	0.015
Interventricular sept(mm)	8.82 ± 0.78	11.63 ± 1.24	<0.001
LV Poster Wall(mm)	8.49 ± 0.82	10.71 ± 0.90	<0.001
EF (%)	60.12 ± 4	62.44 ± 4	0.147

Legend: RMR, resting metabolic rate; RQ, respiratory quotient; BMI, body mass index; WC, Waist circumferences; HC, hip circumferences; TBW, total body water; ECW, extracellular water; FFM, free fat mass; MM, muscle mass; FM, fat mass; T Cholesterol, total cholesterol; HDL, high density lipoprotein; LDL, low density lipoprotein; SBP, systolic blood pressure; DBP, diastolic blood pressure; HR, heart rate; CIMT, Carotid Intima-Media Thickness; LVMI, left ventricular mass; AR, Aortic Root; LAD, Left Atrium Diameter; LV, left ventricular; sept, septum; diam, diameter; EF, Ejection Fraction.

**Table 3:** The area under the receiver operating characteristic (ROC) curve analysis.

Area	Standard Error	*p*	CI 95%	
			Lower Limit	Higher Limit
0.720	0.093	0.031	0.537	0.903

## 4. Discussion

In this study we demonstrated, among subjects with obesity treated with the same dietary and pharmacological interventions, that RQ, an index of nutrient utilization, was significantly different between those participants with and without cardiac remodeling. Moreover, the CIMT was significantly different between participants with and without concentric remodeling and also between participants with and without a high RQ value [5,6].

This is a new, very intriguing, finding. In fact, atherosclerotic CVD is still the leading cause of morbidity and mortality worldwide, although the best possible medical therapy has been prescribed for primary and secondary preventions.

LVCR and CIMT measurements are non-invasive procedures that allow clinicians to predict the future risk of CVD [8,25,26]. These methods are important in individuals with obesity since obesity adversely affects cardiac and vascular function [27]. In the nutritional setting, it has been shown that a low-fat utilization when fasting and the tendency to burn more glucose (suggested by high RQ), is associated with CIMT [7] and risk factors for CVD [5,6]. For this role, the contribution of FAs is central. In fact, oxidation of FAs provides a large amount of the energy required by the heart, due to the intact function of the β-oxidation pathway, the Krebs cycle and the respiratory chain. Defects of fatty acid oxidation are well known disorders associated with cardiomyopathy, individually rare, but collectively frequent due to the number of different enzymes involved [28], confirming how fat metabolism is crucial for a normal heart. Data on substrate metabolism in patients with cardiac remodeling, LVH or congestive heart failure are limited, however, overall it was suggested that in these clinical conditions there is a down regulation of fatty acid oxidation [29]. Alteration in substrates metabolism have been reported in several animal models of LVH and heart failure [30,31]. At themoment, cardiac positron emission tomography (PET) with the use of a metabolic tracer or oxidative metabolism measured by proton MRI spectroscopy have been proposed as non-invasive methods of investigating the myocardial defects of long-chain fatty acid oxidation [32]. Here, we have hypothesized and shown a relationship between cardiac remodeling and shifts in fuel metabolism, from fat to glucose utilization measured with indirect calorimetry in overweight/obese individuals. This association is plausible since heart metabolism at rest completely depends on FAs oxidation coming from circulation [1], thus it is more sensitive to any alteration in substrates utilization. Of course, it is well known that the contribution of the heart metabolism on energy expenditure at rest is limited in comparison to that of liver, brain and skeletal muscle. However the metabolic rate of the heart is about twice the value of liver and brain, and approximately 30 times greater than that of skeletal muscle [33].In addition, several data have shown a linear decline in high-metabolic-rate organ weight with an increasing age for the brain, liver, and kidneys, while the weight for heart increased with age [34]. FAs play also an important roles in the inter-organ communication in lipid metabolism regulation [35]. Consequently, taking into account all these concepts, the identification of a clinical condition in which the overall fat utilization is reduced by indirect calorimetry may reflect the deficiency of fuel utilization in the heart. It is a tool that may be considered as simpler. We believe that, at least in our investigation, RQ measurement may help the interpreting the (patho)physiological mechanisms of fuel utilization in subjects with obesity. In particular, all participants undergo the same dietary and pharmacological interventions but those with LVCR had inability to oxidize fat at fast. It may be possible that some subjects were “low responders” to therapies and consequently undergo both heart and vascular remodeling. In fact, serum triglycerides, as well as uric acid and glucose were different between subjects with and without LVCR nevertheless they followed the same diet. The response to antihypertensive agents could be also different between subjects with and without cardiac remodeling, although the use of medications was not significant different (Table 1). Unfortunately, we can’t address the impact of different dosage of medications. However, the difference in RQ between these two groups of relatively young adults remains and suggests the presence of specific mechanisms of nutrients utilizations linked to the detrimental cardiac chamber remodeling. This result is of great interest since it has been shown, in rats, that phytochemicals found in foods can reverse cardiac remodeling induced by a high-carbohydrate, high-fat diet [36,37]. This effect seems due to an increasing fatty acid oxidation [36]. Thus, our study also has therapeutic implications as we assume that a change in diet in individuals with a high RQ would prevent cardiac remodeling. Several unresolved issues in the practice of indirect calorimetry are highlighted, and it is expected that this study will lead to further work aimed at improving what has become extremely innovative research. It is important to remember that nevertheless single-fasting glucose measurements are subject to substantial within-person variation and many investigations do not perform 2 h glucose tolerance testing, fasting glucose is now commonly measured for early detection of diabetes mellitus and also to suggests information about individual CVD risk [38]. It is well known that also the flow mediated dilation (FMD), a vascular response associated to coronary disease, can be influenced by dietary intake, caffeine, alcohol ingestion and supplement use and time of day at which assessments are made may also affect FMD [39]. Similarly, although different methodological approaches may limit its validity, there are both weaknesses and strengths to pursue the technique to assess RQ in the obese as a physiological research tool.

## 5. Conclusions

RQ measurement may contribute to explain the different mechanisms of fuel utilization in subjects with obesity receiving equal interventions.
